# Importance of global communication to combat global pandemics: Lessons from the HIV Online Provider Education programme

**DOI:** 10.4102/sajhivmed.v22i1.1281

**Published:** 2021-08-31

**Authors:** Efeose A. Airewele, Henry Sunpath, Mahomed-Yunus S. Moosa, Rajesh T. Gandhi

**Affiliations:** 1 Infectious Disease Division, Department of Medicine, Massachusetts General Hospital, Boston, Massachusetts, United States of America; 2 Harvard University Center for AIDS Research, Boston, Massachusetts, United States of America; 3 Department of Infectious Diseases, Nelson R. Mandela School of Medicine, University of KwaZulu-Natal, Durban, South Africa

**Keywords:** HIV, AIDS, COVID-19, COVID, pandemic, communication, collaboration, global health, public health

## Abstract

In many ways, the coronavirus disease 2019 (COVID-19) pandemic mirrors the challenges, lessons and opportunities of the HIV pandemic. In this article, we argue that global pandemics such as COVID-19 and HIV require a global response. We highlight the HIV Online Provider Education (HOPE) programme as an example of the importance of global communication when combating a pandemic. From both the COVID-19 and HIV pandemics, we have learned that to optimise health worldwide, it is necessary to have effective and efficient means of swiftly sharing experiences, expertise, best practices and guidelines. To prepare for the next public health emergency, clinicians and researchers must put in place and promote effective programmes for global communication.

## Twin pandemics: Coronavirus disease 2019 and HIV

When the novel coronavirus emerged in late 2019, much was unknown about its transmission, treatment and trajectory. In response to the rapidly growing case numbers and global spread, the World Health Organization declared the coronavirus disease 2019 (COVID-19) outbreak a public health emergency of international concern on 30 January 2020.^1^ At the time, there was widespread uncertainty and fear about the mode of spread of severe acute respiratory syndrome coronavirus 2 (SARS-CoV-2), leading to hysteria and responses such as washing down groceries and other measures that were ultimately discarded. Early on, there were no known treatments for COVID-19, and in many places around the world, medications that ultimately proved ineffective – like hydroxychloroquine – were frequently administered out of desperation.^2,3^ In addition, it soon became clear that COVID-19 was disproportionately affecting key populations such as racial and ethnic minorities in the United States (US) and other countries and the poor all around the world. In many ways, these early responses to COVID-19 were eerily reminiscent of the world’s first tentative responses to HIV/AIDS (Box 1).

BOX 1 Parallels between the HIV and coronavirus disease 2019 pandemics.

- Initial uncertainty about transmission leading to stigma and ineffective prevention measures
- Prior to definitive clinical trials, desperation leading to the use of unproven and potentially harmful therapies
- Disproportionate impact on key populations, like the poor and racial/ethnic minorities
- Highlights the intertwining of politics and public health: for HIV, initial neglect and a lack of funding; for COVID-19, contradictory evaluations of the severity of the pandemic and mixed messages regarding prevention measures
- Global response required to end the spread of the pandemic
- Importance of global communication and the exchange of experiences by clinicians to develop optimal management strategies
- COVID-19, coronavirus disease 2019.

Similar to the COVID-19 pandemic, when the first patient with AIDS was identified in the early 1980s, there was widespread fear of a new, unknown virus. Because of uncertainties about how HIV was spread, people were shunned and stigmatised. In addition, people with HIV then – and now – often belonged to the most vulnerable and marginalised communities. The parallels between HIV and COVID-19 treatments are also telling. As with COVID-19, early treatments for HIV were largely ineffective and often harmful. By 1987, the United States (US) Food and Drug Administration had approved the first antiretroviral medication, zidovudine, but it soon became evident that single-drug therapy had serious limitations. Since then, significant progress has been made in developing well-tolerated, highly effective combination antiretroviral treatments (ART), resulting in dramatic reductions in both morbidity and mortality.

Despite the similarities, there are also vast differences between COVID-19 and HIV. The former is primarily a respiratory viral infection transmitted by droplets and through the airborne route, with the majority of patients recovering spontaneously. The latter is a sexually transmitted and blood-borne viral disease that causes immunodeficiency and opportunistic conditions and is usually fatal if not treated. The former has had an unprecedented impact on the global economy, in part because of its rapid trajectory necessitating widespread lockdowns. Nevertheless, the parallels summarised above, and the global nature of both pandemics, highlight the critical need for global communication in our response.

A prime example of global communication to advance health occurred during the roll-out of HIV treatment throughout the world. The largest number of people with HIV reside in sub-Saharan Africa, specifically South Africa. In the early days of ART, management of HIV was rapidly evolving, and it was critically important to keep clinicians abreast of the latest in the treatment of this disease. It was in this context that the HIV Online Provider Education (HOPE) programme was developed and now serves as an important example of how a global communication network can be utilised to advance health in disparate parts of the world.

## Development of the HIV Online Provider Education programme

The HOPE programme was established in 2003, around the time of the launch of the President’s Emergency Plan for AIDS Relief (PEPFAR). The HOPE programme was developed by three collaborators: Dr Rajesh Gandhi, Director of HIV Clinical Services and Education at Massachusetts General Hospital, Professor of Medicine at Harvard Medical School and, at the time, Director of the Harvard University Center for AIDS Research Clinical Core; Dr Henry Sunpath, then Chief of Medicine at McCord Hospital; and Dr Yunus Moosa, Chief of Infectious Diseases at the King Edward VIII Hospital, University of KwaZulu-Natal. From the beginning, HOPE and a face-to-face yearly conference called the Annual Workshop on Advanced Clinical Care – AIDS, or AWACC – were joint projects of the McCord Hospital, the University of KwaZulu-Natal, Massachusetts General Hospital and the Harvard University Center for AIDS Research. As a result of Drs Sunpath and Moosa’s collaborative activities with the KwaZulu-Natal Department of Public Health, there were frequent discussions and dynamic exchanges of ideas between the conference organisers and public health officials. Most importantly, unlike some educational activities that have originated in the Global North and spread to the Global South, HOPE was in its essence a joint and highly collaborative project – a digital ‘two-way street’ – that fostered global communication around the treatment of people with HIV.

The early interactions between researchers and clinicians in South Africa and the US exemplify the collaborative nature of the HOPE programme and affiliated activities. In 2002, before the national ART roll-out strategy began in South Africa, McCord Hospital was one of the largest centres providing dedicated HIV care in South Africa, where AIDS denialism existed for many years. Early on, clinicians struggled with developing treatment guidelines to manage adverse events, treatment failure and coinfections within the constraints of a limited ART formulary and laboratory support. In 2003 clinicians and researchers from the US, including Prof. Gerald Friedland from Yale and Prof. Bruce Walker from Harvard Medical School, reached out to leaders at McCord Hospital, resulting in joint site visits by senior infectious disease specialists. Jointly and collaboratively, clinicians and researchers developed clinical algorithms to manage a broad array of diagnostic and therapeutic problems. The infectious diseases unit at the University of KwaZulu-Natal also participated in the joint working group with colleagues from the US and McCord and participated in case-based discussions as the ART programme grew to about 3500 patients. As word of the success of the work at McCord Hospital spread in the community, many clinicians from KwaZulu-Natal and throughout South Africa were keen to collaborate with us to develop ART programmes. In 2004, PEPFAR came on board to support the McCord Hospital programme, and the numbers of patients on ART expanded significantly. In addition, many surrounding clinics sent healthcare workers to our regular continuing medical education meetings. The Department of Health was also interested in working with us to develop treatment protocols and training programmes. With the leading roles of Prof. Gandhi and our team in Durban, we started the first AWACC in 2006. The meeting was widely acclaimed as a most relevant exchange of expertise in HIV care in resource-limited settings in Africa.

## The HIV Online Provider Education programme

The HOPE programme focuses on topics relevant to the care of people with HIV in resource-limited settings. Through regular internet-based conferences, the HOPE programme serves as an opportunity for continuing education and the sharing of best practices in HIV medicine for clinicians worldwide. In addition to a programme focused on physicians, a parallel conference was designed specifically for nurses.^4^

During the early years of the HOPE programme, clinicians in the US had more experience treating patients with ART. Because of this, HOPE conferences were primarily case based and followed the ‘mentoring the mentor’ model to support South African, Zimbabwean, US, Haitian, Dominican and Indian clinicians. It was realised early on that clinicians learned best when clinical problems were constructed around a real-life clinical case, and this led to a case-based as opposed to a didactic approach to teaching.

Initially, because of the toxicities of the available antiretroviral medications, much of the focus was on managing the complications arising from treatment. In particular, the unavoidable reliance on antiretroviral agents such as stavudine and didanosine led to a rise in complex metabolic complications such as lactic acidosis, pancreatitis, lipodystrophy and peripheral neuropathy.^5^ Through the HOPE programme, clinicians gained familiarity with the adverse events commonly associated with ART and developed confidence in identifying and managing these conditions using locally available resources. Physicians in the US offered advice based on years of clinical experience with similar toxicities and often influenced discussions and policies in South Africa. The HOPE conferences and in-person conferences like AWACC also served as opportunities for clinicians to advocate for access to better-tolerated and less toxic medications, like tenofovir. Eliminating stavudine and didanosine from the ART formulary in South Africa took approximately 6 years, during which time physicians worked collaboratively to develop guidelines to manage toxicities.

In addition to toxicities, antiretroviral drug resistance emerged as a significant threat to the impact of these medications in reducing morbidity and mortality. A 2008 study from two clinics in Durban, South Africa, demonstrated that > 83% of patients with virological failure had a least one major resistance mutation, and, of particular concern, mutations resulting in resistance to at least two classes of drugs were present in more than half of those tested.^6^ The HOPE conference served as a critical forum for clinicians to discuss and develop management strategies for patients with drug-resistant HIV. Again, these discussions influenced policies related to the management of virological failure.

Attending the HOPE conferences in real time served as an opportunity for the immediate exchange of knowledge while simultaneously facilitating networking with physicians around the world. Through the HOPE programme, physicians in the US and South Africa have been able to connect and engage in collaborative research. The potential to serve as a networking platform was an unexpected beneficial outcome of the real-time nature of the HOPE conferences. For those unable to join the conferences live, recordings, references and slide presentations have been made available at no cost on the HOPE website after each conference.

What has been the utilisation of and response to HOPE? When HOPE first started, 2000–3000 users logged in annually. In recent years, this number has increased at least threefold to around 9000 attendees per year. Since tracking began in 2012, almost 20 000 users have viewed the conferences, with almost 95 000 conference page views. About 60% of those accessing conferences are medical doctors, 20% are nurses, 10% are nurse practitioners and 10% are medical students. In terms of geographic distribution, visitors are most frequently from the US, South Africa, Dominican Republic, Canada and Argentina.

Surveys of HOPE conference attendees reveal that 95% of the participants report the content is relevant to their practice and that a similar proportion report that there is an appropriate mix of didactic and interactive material. In its early years, attendees asked for additional content relevant to nurses, which led to the development of a parallel HOPE nurses’ conference. The cost of the programme has mainly been the time and effort of the organisers who arrange the speakers and discussants, as well as the project coordinator for the programme, whose time and effort has been supported by the Harvard University Center for AIDS Research as a component of its training mission. The HOPE conference itself is free of charge for all participants.

Over time, as South African clinicians gained experience in the care of patients with HIV, they themselves evolved into experts in their own right, and the HOPE programme shifted focus to summarising the latest in HIV research and innovation. These conferences now serve as an important platform to keep providers in the US, South Africa and other parts of the globe up to date with the latest advances in the field. Of late, the HOPE programme has pivoted to incorporating content geared towards confronting the latest global pandemic, COVID-19. One such recent conference described the impact of the B.1.351 (beta) variant of SARS-CoV-2, which was instrumental in alerting US clinicians to the challenges ahead.

## Looking ahead

Through the HOPE programme, we can see how global communication positively impacts health education internationally. By expanding and developing other programmes like HOPE, clinicians can position themselves to mount an effective global response to health threats like HIV and COVID-19. Interactive online clinical discussions are one of many ways in which the global medical community can bridge the gap in medical education to promote better health outcomes for people everywhere. To reach the Joint United Nations Programme on HIV/AIDS targets for 2030, it is of the utmost importance that collaborative educational initiatives that started at the turn of this century expand and continue.
